# Fresh Meat Classification Using Laser-Induced Breakdown Spectroscopy Assisted by LightGBM and Optuna

**DOI:** 10.3390/foods13132028

**Published:** 2024-06-26

**Authors:** Kaifeng Mo, Yun Tang, Yining Zhu, Xiangyou Li, Jingfeng Li, Xuxiang Peng, Ping Liao, Penghui Zou

**Affiliations:** 1 Hunan Province Key Laboratory of Intelligent Sensors and Advanced Sensor Materials, School of Physics and Electronics Science, Hunan University of Science and Technology, Xiangtan 411201, China; mkf@mail.hnust.edu.cn (K.M.); jingfengli@mail.hnust.edu.cn (J.L.); 21020802001@mail.hnust.edu.cn (X.P.); 22010801001@mail.hnust.edu.cn (P.L.); 22020804012@mail.hnust.edu.cn (P.Z.); 2 Wuhan National Laboratory for Optoelectronics (WNLO), Huazhong University of Science and Technology, Wuhan 430074, China; zyn_huster@126.com (Y.Z.);

**Keywords:** laser-induced breakdown spectroscopy, LightGBM, optuna, meat classification

## Abstract

To enhance the accuracy of identifying fresh meat varieties using laser-induced breakdown spectroscopy (LIBS), we utilized the LightGBM model in combination with the Optuna algorithm. The procedure involved flattening fresh meat slices with glass slides and collecting spectral data of the plasma from the surfaces of the fresh meat tissues (pork, beef, and chicken) using LIBS technology. A total of 900 spectra were collected. Initially, we established LightGBM and SVM (support vector machine) models for the collected spectra. Subsequently, we applied information gain and peak extraction algorithms to select the features for each model. We then employed Optuna to optimize the hyperparameters of the LightGBM model, while a 10-fold cross-validation was conducted to determine the optimal parameters for SVM. Ultimately, the LightGBM model achieved higher accuracy, macro-F1, and Cohen’s kappa coefficient (kappa coefficient) values of 0.9370, 0.9364, and 0.9244, respectively, compared to the SVM model’s values of 0.8888, 0.8881, and 0.8666. This study provides a novel method for the rapid classification of fresh meat varieties using LIBS.

## 1. Introduction

Meat serves as a crucial source of animal protein in the human diet. In today’s world of increasing food consumption, consumers are increasingly demanding the safety and quality of meat products. However, as the consumption of meat products continues to escalate, the issue of adulteration in these products has emerged as a significant concern for food safety. Some enterprises substitute relatively inexpensive meat for more expensive kinds for sale, which is one of the most common practices. This not only deceives consumers but also poses potential health risks, as adulterated meats often bypass essential inspection and quarantine processes, which increases the possibility of carrying harmful bacteria, viruses, and other microorganisms. Meat products carrying allergens may trigger severe allergic reactions. Moreover, the fraudulent substitution of meat types may have religious or cultural impacts on individuals whose dietary laws prohibit the consumption of certain meats. For instance, the inadvertent consumption of pork, when it is mislabeled as beef or chicken, can cause distress among groups for whom pork consumption is forbidden for religious reasons. Furthermore, the introduction of undeclared additives or fillers to increase the volume of meat products adds another layer of risk. These substances may range from water and fat to more harmful chemical fillers, potentially exposing consumers to unknown allergens and chemical contaminants. Consequently, there is a pressing need for fast and accurate meat product classification and analysis to promote standardization in the meat market and ensure food safety. Following the horse meat scandal that erupted in Europe in 2013 [1], research on meat adulteration and fraud has significantly increased. Multiple techniques have been applied to identify meat types. Traditional techniques for meat classification encompass capillary gel electrophoresis [2], polymerase chain reaction [3], gas chromatography mass spectrometry [4], polymerase chain reaction [5], DNA barcoding [6], among others. Due to superior instrumental features, the protocols based on mass spectrometry [7] are an important method for food adulteration. Anjar Windarsih [8] et al. conducted untargeted metabolomics and proteomics using liquid chromatography–high resolution mass spectrometry (LC-HRMS) to detect pork adulteration in Pangasius hypophthalmus meat (PHM). They successfully used principal components analysis (PCA) and partial least squares discriminant analysis (PLS-DA) to distinguish authentic and adulterated PHM with fitness (R > 0.95) and predictivity (Q > 0.5). Yingying Zhang et al. [9] identified and quantified fox meat in meat products by liquid chromatography–tandem mass spectrometry (LC-MS/MS). Sara W. Erasmus et al. [10] utilized proton-transfer reaction mass spectrometry (PTR-MS) and PLS-DA to distinguish lamb and fat from different regions. They used four different PLS-DA models that take the full mass spectra as input, identifying the lamb and fat samples into “origin” (six different regions) and “provenance” (Karoo vs. non-Karoo) groups. Keyuan Pu1 [11] et al. used matrix-assisted laser desorption/ionization time-of-flight mass spectrometry (MALDI-TOF MS) protein profiling combined with PLS-DA for beef adulteration. They achieved an average prediction accuracy of 94.7% through blind tests. Although mass spectrometry has the advantages of high sensitivity, accuracy, and precision, it necessitates specialized personnel to conduct the tests and suffers from drawbacks such as complex operations, time-consuming procedures, the need for expensive instruments, and intricate processes. These limitations fail to meet the demand for rapid detection. Thus, there is an urgent requirement for a simple and expeditious detection method. 

Laser-induced breakdown spectroscopy (LIBS) is a promising technique in the field of atomic emission spectroscopy [12]. The LIBS technique utilizes high-power laser pulses focused on the sample surface to generate plasma, which emits a spectrum as it decays, and this spectrum carries information about the chemical composition of the sample. By analyzing this spectrum, the types and quantities of elements in the sample can be determined. It offers several advantages, including rapid detection [13], micro-destruction of the sample [14], simple or no sample preparation [15], and the ability to perform remote detection [16,17]. As a result, LIBS has found widespread applications in various domains such as industrial manufacture [18], food safety [19,20,21], environmental monitoring [22], biomedical research [23,24], and even space exploration [25]. Recently, there has been growing interest in integration of LIBS technology with chemometric methods for the identification of biological tissues. Bilge et al. [26] employed LIBS in combination with PCA to classify meat products, specifically pork, beef, and chicken, after crushing and pressing. They achieved a recognition rate of 83.37%. Additionally, they conducted partial least squares (PLS) quantitative analysis on adulterated meat products. Casado-Gavalda et al. [27] utilized LIBS to detect copper in beef offal, enabling the determination of its degradation. Sezer et al. [28] applied LIBS for the identification of milk fraud. Chu et al. [29] applied multiplicative scatter correction (MSC) to first preprocess the spectrum for the correction of spectrum scatter and improve spectral stability and inputted the corrected spectra into the K-nearest neighbor (KNN) for classification. Ultimately, they improved the accuracy of meat classification and the stability of the spectrum. Velioglu et al. [30] used PCA and PLS for the analysis of the collected LIBS spectra to identify adulteration in beef and to carry out quantitative analysis on beef adulteration. The coefficient of determination (R^2^) was 0.947 and the limit of detection values was 3.8% for adulterated beef samples. Sezer et al. [31] adopted a protein-based LIBS method combined with PCA and PLS to discriminate among three meat species (beef, chicken, and pork). The limit of detection values of beef adulteration with chicken and pork were 2.84% and 3.89%. These studies demonstrate the potential of LIBS technology in the field of meat classification and analysis, highlighting its capability to provide rapid and accurate results in various applications. Compared to the aforementioned analytical algorithms used in LIBS, this study employs Light Gradient Boosting Machine (LightGBM), a novel Gradient Boosting Decision Tree (GBDT) algorithm. LightGBM significantly outperforms conventional GBDT algorithms in computation speed and memory consumption without compromising accuracy. LightGBM offers several advantages, including its resistance to overfitting, fast training speed with large sample sizes, and more. It has found widespread applications in various fields such as the financial industry, biomedical research [32], and environmental studies [33]. However, the LightGBM algorithm has never been applied in the field of LIBS before.

In this work, we utilized the glass-pressed slice method for sample preparation. We employed LightGBM and SVM (support vector machine) to classify the spectra corresponding to six different kinds of meat, including pork, beef, and chicken, collected by LIBS, aiming for quick and accurate classification. We optimized the hyperparameters of LightGBM using Optuna to enhance the accuracy of the classification model. Finally, we compared the recognition accuracies of the two classification models, LightGBM and SVM, confirming that LightGBM outperforms in recognizing fresh meat varieties. This study introduces a novel approach for meat classification in the field of LIBS.

## 2. Experiments

### 2.1. Sample Preparation

The fresh meat samples utilized in this experiment encompass Enshi earth pork loin (abbreviated as Enshi pork), black pork loin (abbreviated as black pork), COFCO (China Oil & Foodstuffs Corporation, Beijing, China) pork, sirloin beef, silverside beef, and chicken breast. The experimental samples were purchased from local supermarkets, and are all meats that people commonly buy in daily life. The purchased fresh meat samples were sliced into small pieces of approximately 50 mm × 18 mm in length and width and 2–3 mm in thickness, placed flat on a glass slide (25.4 mm × 76.2 mm), and pressed with another glass slide. The other glass slide was pressed on top of the meat slice and held down for 1 min with a 10 kg weight so that the meat slice lay flat on the slide below it, and the top glass slide was removed after pressing. The samples processed by this method had a large and flat surface. 

### 2.2. Experimental Setup and Measurement

**Figure 1:**
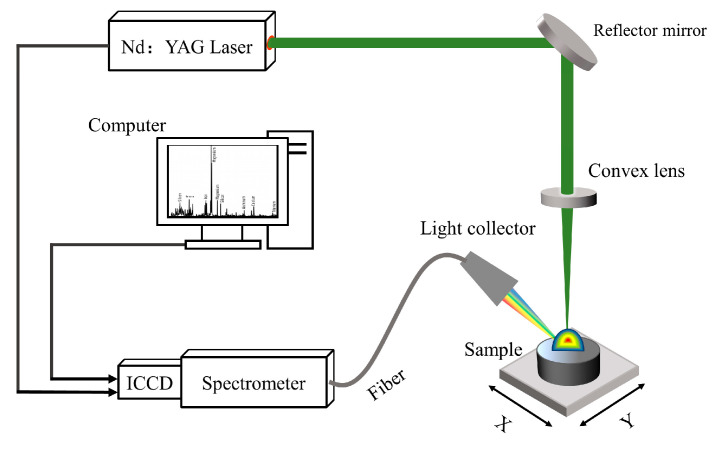
Schematic diagram of the experimental setup.

The experimental setup of the LIBS detection system used in this experiment is illustrated in Figure 1. A Q-switched Nd:YAG pulsed laser (Quantel, Brilliant B, Les Ulis, France) with a wavelength of 532 nm, pulse width of 8 ns, and maximum repetition frequency of 10 Hz was employed. The laser beam was focused onto the sample surface through a reflector and a flat convex lens (with a focal length of 100 mm) to generate a plasma emission for spectral analysis. The emitted plasma radiation was collected by a light collector, coupled into a UV-enhanced optical fiber with a diameter of 100 μm, and transmitted to a spectrometer (Andor technology, ME 5000, resolution λ/Δλ= 5000, wavelength range 200–950 nm) equipped with an intensified charge-coupled device (ICCD) camera (Andor technology, Belfast, UK, iStarDH-334T, 1024 × 1024 pixels) for spectral recording. The acquired data were subsequently analyzed and processed using a computer. To avoid repetitive laser pulse impacts on the same position of the sample, the experimental samples were positioned on a two-dimensional displacement stage. In this experiment, the displacement platform executed a scanning motion in a bow-shaped pattern.

**Figure 2:**
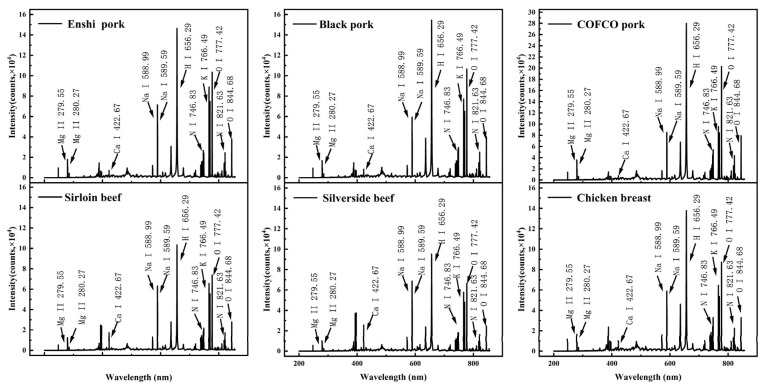
The full spectra of the six samples.

The experiment utilized optimized parameters, including an energy of 30 mJ/pulse and a frequency of 5 Hz. For the accumulation mode of the spectrometer, the acquisition parameters were as follows: an acquisition delay time of 0.9 μs, and a gate width of 10 μs. Additionally, 10 laser pulses were accumulated per spectrum. A total of five samples for each category of fresh meat were pressed. For each sample, 30 spectra were acquired, resulting in a comprehensive dataset of 900 spectra of the six fresh meat tissue samples. Figure 2 displays the full spectra of the six samples. It can be observed that each meat category contains elements such as Mg, Ca, Na, K, N, and O. However, visual differentiation between the various meat tissues based on the full spectra is challenging. Therefore, employing classification algorithms is crucial to differentiate between different meat categories.

## 3. Method

### 3.1. LightGBM

Gradient Boosting Decision Tree (GBDT) is a widely used machine learning model employed in various tasks, including classification, regression, and ranking. Given a training dataset $S\;={\left\{x_{i},y_{i}\right\}}_{i=1}^{n}$, where x is the data sample and y is the label. The objective of GBDT is to find an approximation $\hat{f}(x)$ that minimizes the expected value of a specific loss function $L\left(y,f\left(x\right)\right)$, which can be formulated as follows:

(1) $$\hat{f}=arg\min E_{x,y}\left[L\left(y,f\left(x\right)\right)\right]$$

The loss function $L\left(y,f\left(x\right)\right)$ is the difference between the final predictor variables and the actual variables after m iterations. After m iterations, GBDT incorporates m weak classifiers into the final model, each with its respective weights:

(2) $$f_{m}\left(x\right)=f_{m-1}\left(x\right)+\rho_{m}h_{m}$$

where $\rho_{m}=\underset{\rho }{arg\min}\sum_{i=1}^{n}L\left(y_{i},f_{m-1}\left(x_{i}\right)+\rho h_{m}\left(x_{i}\right)\right)$ is the weight of the $m^{th}$ function and $h_{m}\left(x\right)$. $h_{m}\left(x\right)$ is the base decision tree.

However, traditional GBDT faces challenges in achieving satisfactory results in terms of both efficiency and accuracy when dealing with high-dimensional features and large datasets. One major reason is that GBDT’s weak classifiers, typically based on decision trees, require calculating the information gain for each feature to find the best split points, and this process can be highly time-consuming. To address this issue, Microsoft and Peking University proposed LightGBM [34] in 2017, which is a novel GBDT implementation incorporating gradient-based one-side sampling (GOSS) and exclusive feature bundling (EFB). Additionally, the histogram-based algorithm serves as the foundation for GOSS and EFB.

GOSS is a novel sampling technique that effectively reduces computational scale while preserving training accuracy. The process involves several steps. First, the training instances are sorted in descending order based on the absolute values of their gradients. Next, the top  a×100% instances with the larger gradients are selected as a subset, A. Then, randomly sample a subset, B, with a size of $b\times A^{c}$ from the remaining set $A^{c}$ consisting of $\left(1-a\right)\times 100\%$ instances with smaller gradients. Finally, the instances are split based on the estimated variance gain ${\tilde{V}}_{j}\left(d\right)$ on A∪B, i.e.,

(3) $${\tilde{V}}_{j}\left(d\right)=\frac{1}{n}\left(\frac{{\left(\sum_{x_{i}\in A_{l}}g_{i}+\frac{1-a}{b}\sum_{x_{i}\in_{B_{l}}}g_{i}\right)}^{2}}{n_{l}^{j}\left(d\right)}+\frac{{\left(\sum_{x_{i}\in A_{r}}g_{i}+\frac{1-a}{b}\sum_{x_{i}\in_{B_{r}}}g_{i}\right)}^{2}}{n_{r}^{j}\left(d\right)}\right)$$

where $A_{l}=\left\{x_{i}\in A:x_{ij}\leq d\right\}$, $A_{r}=\left\{x_{i}\in A:x_{ij}>d\right\}$, $B_{l}=\left\{x_{i}\in B:x_{ij}\leq d\right\}$, $B_{r}=\left\{x_{i}\in B:x_{ij}>d\right\}$. l and r represents the left and right subtrees of the decision tree. d represents the threshold for decision tree splitting. j is the feature. $g_{i}$ represents the negative gradient of the loss function in each iteration of gradient boosting. The coefficient $\frac{1-a}{b}$ is applied to normalize the sum of the gradients. 

The EFB algorithm reduces computation costs by combining multiple exclusive features into fewer dense features. In high-dimensional data, sparsity is common, resulting in many exclusive features. EFB addresses this by treating mutually exclusive bundled features as a single feature, thus reducing the number of features. By employing a feature scanning algorithm, LightGBM constructs feature histograms from these feature bundles. 

Furthermore, unlike most GBDT implementations that use a level-wise (depth) approach for growing decision trees, LightGBM adopts a leaf-wise (best-first) strategy. This change reduces losses when growing the same number of leaves. However, when dealing with small datasets, the leaf-wise approach may result in overfitting. To address this, LightGBM provides the option to set the max_depth parameter, which limits the tree depth and helps prevent overfitting. Table 1 presents the key parameters of LightGBM.

### 3.2. Optuna

Hyperparameter optimization is a critical and complex task in GBDT. The performance and the output of the model heavily depend on the optimization of hyperparameters. However, the GDBT algorithm has an extensive set of hyperparameters, making it challenging to manually select or randomly search for the best parameters. This often leads to unsatisfactory results and wastes time and effort. To address this, we utilized Optuna [35], a hyperparameter optimization framework, to automate the tuning process. Optuna offers several key features, including:

- Define-by-run style API: Optuna provides a flexible API (application programming interface) that allows defining and optimizing hyperparameters within the code, making it easy to incorporate into workflows.
- Efficient sampling and pruning mechanism: Optuna employs efficient sampling and pruning techniques to explore the hyperparameter search space effectively and eliminate unpromising trials, thus improving efficiency.
- Easy to setup: Optuna is designed to be user-friendly and easy to set up, enabling users to quickly get started with hyperparameter optimization.

In Optuna, the concept of “define-by-run” refers to the ability for users to dynamically construct the search space during runtime. Optuna formulates the hyperparameter optimization process as the maximization of the minimization of an objective function’s score. Each iteration of the objective function takes a set of hyperparameters, calculates a score, and represents each optimization process as a study, while each evaluation of the objective function is referred to as a trial. By utilizing the objective function, Optuna enables the dynamic construction of the search space without relying on externally defined static values.

Optuna offers both relational sampling and independent sampling approaches. It provides various independent sampling methods such as the tree-structured Parzen estimator (TPE) and related algorithms like the covariance matrix adaptation evolution strategy (CMA-ES). Additionally, Optuna supports users in using their customized sampling methods. A well-designed pruning algorithm can effectively reduce the time required for optimization. In Optuna, the “report API” is used to monitor the objective values in each trial, while the “should_prune API” is invoked to determine whether it is necessary to terminate unpromising trials. Optuna is commonly used in the following steps:

(1) Define an objective function that takes a set of hyperparameters as input and returns the metrics representing model performance (such as the accuracy of the validation set, root mean square error (RMSE), and multi-loss). Additionally, specify the range of hyperparameters that need to be adjusted, including the distribution type and value range for each hyperparameter.
(2) Create an Optuna study to minimize or maximize the objective function and set the number of trials in a study. In each trial, Optuna finds a set of hyperparameters and passes them into the objective function. The sampling methods were used to traverse the hyperparameter space.
(3) Get the result, the best hyperparameter combination at the end of all trials. The “plot_optimization_history (study) API” can be used to observe the trend of the objective function’s value increasing or decreasing.
(4) Apply the optimal hyperparameters to the classification model and test it on the test set and determine if further optimization is needed.

## 4. Result and Discussion

The datasets were randomly split into training sets and test sets using a ratio of 7:3. The data splitting process was implemented using Python version 3.10. Before the classification algorithm was applied, the LIBS spectra were standardized. The formula for standardization is as follows:

(4) $$x^{*}=\frac{x-\mu }{\sigma }$$

where x represents the sample data, μ represents the mean of the sample data, and σ represents the standard deviation of the sample data. $x^{*}$ is the standardized data.

### 4.1. Classification with SVM

In the task of LIBS data classification, SVM has been widely utilized. For our study, we selected the radial basis function (RBF) kernel for SVM and employed a 10-fold cross-validation method to optimize the parameters. The optimal parameters C and gamma were found to be 512 and 0.001, respectively.

**Figure 3:**
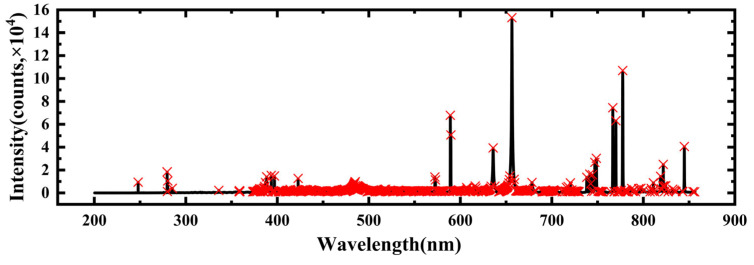
The results of peak detection.

To prepare the input for the SVM training model, we employed a peak extraction algorithm. In order to capture more meaningful information while reducing redundant features, we computed the average spectrum by averaging all spectra from the six categories of samples. The effective spectral features were then obtained through peak extraction from the average spectrum. The results of the peak detection process are illustrated in Figure 3, where the black line represents the mean spectrum and the red crosses indicate the positions of the detected peaks. A total of 712 peak features were identified, and these features were used as inputs for the SVM model trained with the optimal parameters obtained through cross-validation.

**Figure 4:**
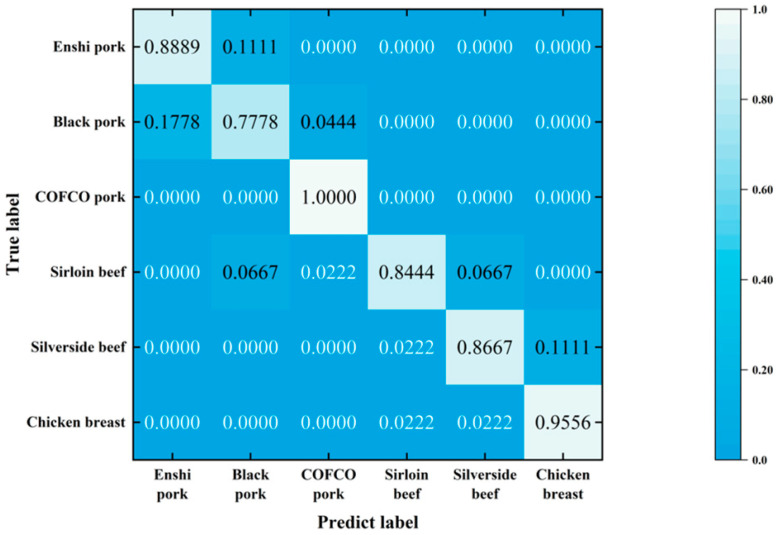
The confusion matrix of the SVM model.

The classification results are illustrated in Figure 4. The accuracy rates for each meat species—Enshi pork, black pork, COFCO pork, sirloin beef, silverside beef, and chicken breast—were 88.89%, 77.78%, 100%, 84.44%, 86.67%, and 95.56%, respectively. The average accuracy across all six categories of meats was 88.89%. Black pork exhibited poor categorization performance, and 17.8% of the samples identified as Enshi pork were actually black pork. On the other hand, COFCO pork demonstrated excellent categorization, achieving a 100% accuracy rate.

### 4.2. Classification with LightGBM

**Figure 5:**
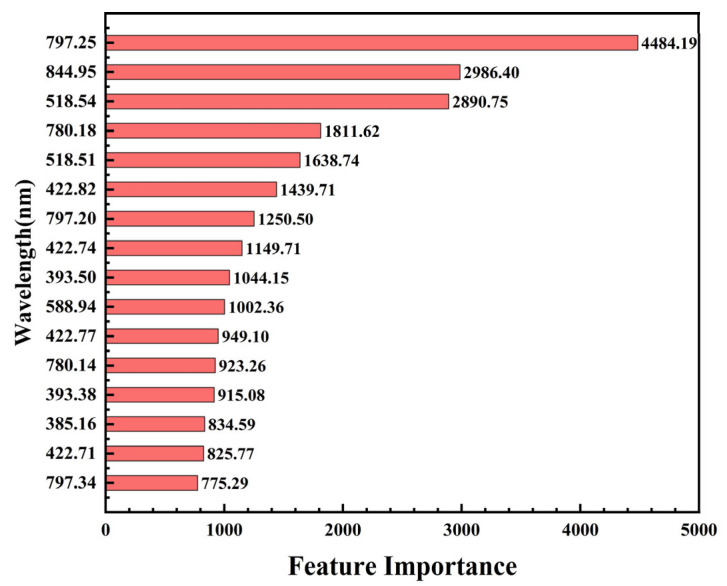
The feature importance scores given by LightGBM.

To enhance the stability and generalization capability of the model, we employed a 10-fold cross-validation method during the training of the LightGBM model. Additionally, we utilized the built-in “feature_importance” API of LightGBM, using the gain metric for calculating feature importance. Gain represents the amount of information that a feature contributes to the classification system. The higher the gain, the more crucial the feature is considered to be. Figure 5 presents the top 16 most important spectral lines based on their calculated importance. The *Y*-axis represents the wavelength of the spectral line. Spectral lines with an importance value greater than 120 were selected as a characteristic spectral line and for input into the LightGBM model, resulting in a total of 91 spectral features being included. This selection was based on both the importance values and the significance of the features in contributing to the classification task.

**Figure 6:**
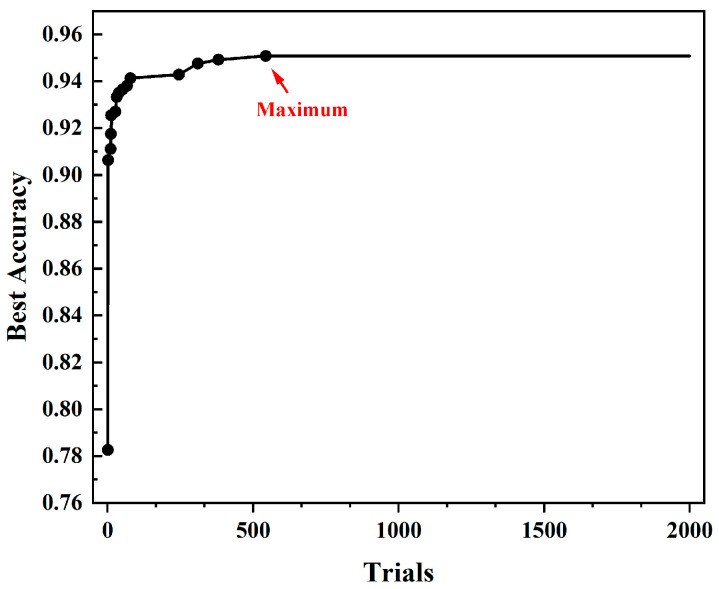
The process of iteratively increasing the test set accuracy during the Optuna optimization.

Before commencing the training of the LightGBM model, we performed a hyperparameter optimization using the Optuna framework. We set up 2000 trials with the suggested method, allowing Optuna to select a set of hyperparameters for training in each trial. The objective function utilized the accuracy on the test set as the score to be maximized. The aim of each trial was to maximize the accuracy of the validation set. Figure 6 illustrates the process of the 2000 trials. As the number of trials increased, the accuracy gradually improved and reached its peak at the 544th trial. However, subsequent trials did not yield further improvement in accuracy. Therefore, we selected the hyperparameters from the 544th trial as the optimized result of the hyperparameter optimization process. The maximum accuracy achieved was 95.07%.

Subsequently, we applied the best model to the test set for recognition, resulting in an average accuracy of 92.22%. However, we observed signs of overfitting in the model. The accuracy of the training set was higher than that of the test set. To address this issue, we conducted further adjustments by optimizing the “min_data_in_leaf”, “bagging_fraction”, and “feature_fraction” hyperparameters through 800 additional trials. Through this optimization process, we obtained optimal values for these hyperparameters: “min_data_in_leaf = 26”, “bagging_fraction = 0.64”, and “feature_fraction = 0.13”. As a result, the average accuracy of the test set increased to 93.70%. These adjustments helped mitigate overfitting, thus leading to improved performance. Table 2 provides a summary of the final optimized hyperparameters and their corresponding optimal values.

**Figure 7:**
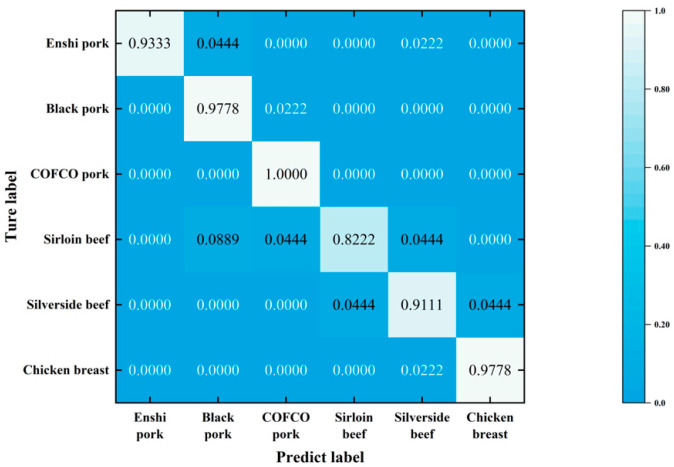
The confusion matrix of the LightGBM model.

After completing the optimization process, we utilized the model with the optimized hyperparameters to recognize the test set. The resulting confusion matrix is depicted in Figure 7. The accuracy rates for each meat species—Enshi pork, black pork, COFCO pork, sirloin beef, silverside beef, and chicken breast—were as follows: 93.33%, 97.78%, 100%, 82.22%, 91.11%, and 97.78%. Notably, the LightGBM model outperformed the SVM model significantly in the classification of black pork. Sirloin beef exhibited poor categorization performance, with 8.89% wrongly identified as black pork, 4.4% as COFCO pork, and 4.44% as silverside beef. The accuracy of sirloin beef classification was decreased compared to the SVM method, which indicates that the selected features of sirloin beef overlapped with those of other meat kinds. Overall, the LightGBM model achieved an average accuracy of 93.7%, surpassing the accuracy obtained by the SVM model.

Finally, Cohen’s kappa coefficient (kappa coefficient) and the macro-F1 score were employed for the final evaluation of the model. The formula for calculating the kappa coefficient is as follows:

(5) $$kappa=\frac{n\times n\times p_{0}-\sum_{i}^{C}a_{i}\times b_{i}}{n\times n-\sum_{i}^{C}a_{i}\times b_{i}}$$

where $p_{0}$ represents the overall classification accuracy, $a_{i}$ is the number of true samples for each category, $b_{i}$ is the number of predicted samples for each category, C is the number of categories, and n is the number of samples.

The formula for calculating the macro-F1 is as follows:

(6) $$Precision_{i}=\frac{TP_{i}}{TP_{i}+FP_{i}}$$

(7) Recalli=TPiTPi+FNi

(8) Macro−F1i=1C∑iC2×Precisioni×RecalliPrecisioni+Recalli

where TP represents the true predictions of the positive samples, FP represents the false predictions of the positive samples, TN represents the true predictions of the negative samples, FN represents the false predictions of the negative samples, i represents the class label, and C represents the total number of classes.

The calculation results of the kappa coefficient and macro-F1 are presented in Table 3. The kappa coefficient of the LightGBM model was higher than that of SVM and closer to 1, indicating the better classification consistency of the LightGBM model.

## 5. Conclusions

In this study, we utilized Optuna for the selection of hyperparameters in the LightGBM model. The best-performing model was then employed to analyze and process the spectra obtained from the LIBS system. Comparing the results with the traditional SVM algorithm, we observed a significant improvement in accuracy with an increase of 4.5%. Additionally, the macro-F1 and kappa coefficients also demonstrated an improvement. These findings highlight the potential of combining LIBS with Optuna and LightGBM algorithms for the rapid detection of fresh meat species. This approach introduces a novel method for the rapid identification of fresh meat in markets, offering promising applications in the field.

## Figures and Tables

**Table 1:** The main parameters of LightGBM.

Parameters	Interpretation
num_leaves	This parameter determines the number of leaves per tree.
learning_rate	This parameter controls the speed of iterations in the training process.
max_depth	This parameter sets the maximum depth of the tree.
min_data	This parameter defines the minimum number of records that a leaf node should have.
feature_fraction	This parameter specifies the fraction of features to be selected at each iteration.
bagging_fraction	This parameter determines the fraction of data to be used for each iteration through bagging.

**Table 2:** The optimal values of LightGBM.

Parameters	Optimal Values
num_leaves	34
learning_rate	0.05343712612981269
max_depth	8
min_data_in_leaf	26
feature_fraction	0.13
bagging_fraction	0.64
max_bin	213
lambda_l1	0
lambda_l2	0
min_gain_to_split	0.005296940015136468

**Table 3:** Performance comparison of the SVM and LightGBM models.

Model	Kappa	Macro-F1	Accuracy
SVM	0.8666	0.8881	88.88%
LightGBM	0.9244	0.9364	93.70%

## Data Availability

The datasets generated for this study are available on request from the corresponding author.

## References

[1] Boyacı İ.H., Temiz H.T., Uysal R.S., Velioğlu H.M., Yadegari R.J., Rishkan M.M. (2014). A novel method for discrimination of beef and horsemeat using Raman spectroscopy. Food Chem..

[2] Vallejo-Cordoba B., González-Córdova A.F., Mazorra-Manzano M.A., Rodríguez-Ramírez R. (2005). Caillary electrophoresis for the analysis of meat authenticity. J. Sep. Sci..

[3] Rahman M.M., Ali M.E., Hamid S.B.A., Mustafa S., Hashim U., Hanapi U.K. (2014). Polymerase chain reaction assay targeting cytochrome b gene for the detection of dog meat adulteration in meatball formulation. Meat Sci..

[4] Nurjuliana M., Che Man Y.B., Mat Hashim D., Mohamed A.K.S. (2011). Rapid identification of pork for halal authentication using the electronic nose and gas chromatography mass spectrometer with headspace analyzer. Meat Sci..

[5] Floren C., Wiedemann I., Brenig B., Schütz E., Beck J. (2015). Species identification and quantification in meat and meat products using droplet digital PCR (ddPCR). Food Chem..

[6] Hellberg R.S., Hernandez B.C., Hernandez E.L. (2017). Identification of meat and poultry species in food products using DNA barcoding. Food Control.

[7] Ivanova B. (2024). Special Issue with Research Topics on “Recent Analysis and Applications of Mass Spectra on Biochemistry”. Int. J. Mol. Sci..

[8] Windarsih A., Suratno, Warmiko H.D., Indrianingsih A.W., Rohman A., Ulumuddin Y.I. (2022). Untargeted metabolomics and proteomics approach using liquid chromatography-Orbitrap high resolution mass spectrometry to detect pork adulteration in Pangasius hypopthalmus meat. Food Chem..

[9] Zhang Y., Liu M., Wang S., Kang C., Zhang M., Li Y. (2022). Identification and quantification of fox meat in meat products by liquid chromatography–tandem mass spectrometry. Food Chem..

[10] Erasmus S.W., Muller M., Alewijn M., Koot A.H., van Ruth S.M., Hoffman L.C. (2017). Proton-transfer reaction mass spectrometry (PTR-MS) for the authentication of regionally unique South African lamb. Food Chem..

[11] Pu K., Qiu J., Li J., Huang W., Lai X., Liu C., Lin Y., Ng K.-M. (2023). MALDI-TOF MS Protein Profiling Combined with Multivariate Analysis for Identification and Quantitation of Beef Adulteration. Food Anal. Methods.

[12] Zou Z., Deng Y., Hu J., Jiang X., Hou X. (2018). Recent trends in atomic fluorescence spectrometry towards miniaturized instrumentation-A review. Anal. Chim. Acta.

[13] Vu D.M., Auxier J.D., Judge E.J., Aldrich K.E., Gifford B.J., Saumon D., Neukirch A.J., Auxier J.P., Barefield J.E., Clegg S.M. (2023). A data analysis method to rapidly characterize gallium concentration in plutonium matrices using LIBS. Spectrochim. Acta Part B At. Spectrosc..

[14] Pagnotta S., Lezzerini M., Ripoll-Seguer L., Hidalgo M., Grifoni E., Legnaioli S., Lorenzetti G., Poggialini F., Palleschi V. (2017). Micro-Laser-Induced Breakdown Spectroscopy (Micro-LIBS) Study on Ancient Roman Mortars. Appl. Spectrosc..

[15] Girón D., Delgado T., Ruiz J., Cabalín L.M., Laserna J.J. (2018). In-situ monitoring and characterization of airborne solid particles in the hostile environment of a steel industry using stand-off LIBS. Measurement.

[16] Guo L.B., Zhu Z.H., Li J.M., Tang Y., Tang S.S., Hao Z.Q., Li X.Y., Lu Y.F., Zeng X.Y. (2018). Determination of boron with molecular emission using laser-induced breakdown spectroscopy combined with laser-induced radical fluorescence. Opt. Express.

[17] Guo Y.M., Guo L.B., Hao Z.Q., Tang Y., Ma S.X., Zeng Q.D., Tang S.S., Li X.Y., Lu Y.F., Zeng X.Y. (2018). Accuracy improvement of iron ore analysis using laser-induced breakdown spectroscopy with a hybrid sparse partial least squares and least-squares support vector machine model. J. Anal. At. Spectrom..

[18] Garcia J.A., da Silva J.R.A., Pereira-Filho E.R. (2021). LIBS as an alternative method to control an industrial hydrometallurgical process for the recovery of Cu in waste from electro-electronic equipment (WEEE). Microchem. J..

[19] Silva T.V., Milori D.M.B.P., Neto J.A.G., Ferreira E.J., Ferreira E.C. (2019). Prediction of black, immature and sour defective beans in coffee blends by using Laser-Induced Breakdown Spectroscopy. Food Chem..

[20] Tian Y., Chen Q., Lin Y., Lu Y., Li Y., Lin H. (2021). Quantitative determination of phosphorus in seafood using laser-induced breakdown spectroscopy combined with machine learning. Spectrochim. Acta Part B At. Spectrosc..

[21] Yang P., Zhou R., Zhang W., Yi R., Tang S., Guo L., Hao Z., Li X., Lu Y., Zeng X. (2019). High-sensitivity determination of cadmium and lead in rice using laser-induced breakdown spectroscopy. Food Chem..

[22] Viana L.F., Súarez Y.R., Cardoso C.A.L., Lima S.M., Andrade L.H.d.C., Lima-Junior S.E. (2019). Use of fish scales in environmental monitoring by the application of Laser-Induced Breakdown Spectroscopy (LIBS). Chemosphere.

[23] Gaudiuso R., Ewusi-Annan E., Melikechi N., Sun X., Liu B., Campesato L.F., Merghoub T. (2018). Using LIBS to diagnose melanoma in biomedical fluids deposited on solid substrates: Limits of direct spectral analysis and capability of machine learning. Spectrochim. Acta Part B At. Spectrosc..

[24] Skalny A.V., Korobeinikova T.V., Aschner M., Baranova O.V., Barbounis E.G., Tsatsakis A., Tinkov A.A. (2023). Medical application of laser-induced breakdown spectroscopy (LIBS) for assessment of trace element and mineral in biosamples: Laboratory and clinical validity of the method. J. Trace Elem. Med. Biol..

[25] Beck P., Meslin P.Y., Fau A., Forni O., Gasnault O., Lasue J., Cousin A., Schröder S., Maurice S., Rapin W. (2024). Detectability of carbon with ChemCam LIBS: Distinguishing sample from Mars atmospheric carbon, and application to Gale crater. Icarus.

[26] Bilge G., Velioglu H.M., Sezer B., Eseller K.E., Boyaci I.H. (2016). Identification of meat species by using laser-induced breakdown spectroscopy. Meat Sci..

[27] Casado-Gavalda M.P., Dixit Y., Geulen D., Cama-Moncunill R., Cama-Moncunill X., Markiewicz-Keszycka M., Cullen P.J., Sullivan C. (2017). Quantification of copper content with laser induced breakdown spectroscopy as a potential indicator of offal adulteration in beef. Talanta.

[28] Sezer B., Durna S., Bilge G., Berkkan A., Yetisemiyen A., Boyaci I.H. (2018). Identification of milk fraud using laser-induced breakdown spectroscopy (LIBS). Int. Dairy J..

[29] Chu Y.W., Tang S.S., Ma S.X., Ma Y.Y., Hao Z.Q., Guo Y.M., Guo L.B., Lu Y.F., Zeng X.Y. (2018). Accuracy and stability improvement for meat species identification using multiplicative scatter correction and laser-induced breakdown spectroscopy. Opt. Express.

[30] Velioglu H.M., Sezer B., Bilge G., Baytur S.E., Boyaci I.H. (2018). Identification of offal adulteration in beef by laser induced breakdown spectroscopy (LIBS). Meat Sci..

[31] Chen C., Zhang Q., Ma Q., Yu B. (2019). LightGBM-PPI: Predicting protein-protein interactions through LightGBM with multi-information fusion. Chemom. Intell. Lab. Syst..

[32] Sezer B., Bjelak A., Velioglu H.M., Boyaci I.H. (2021). Protein based evaluation of meat species by using laser induced breakdown spectroscopy. Meat Sci..

[33] Wei J., Li Z., Pinker R.T., Wang J., Sun L., Xue W., Li R., Cribb M. (2021). Himawari-8-derived diurnal variations in ground-level PM2.5 pollution across China using the fast space-time Light Gradient Boosting Machine (LightGBM). Atmos. Chem. Phys..

[34] Ke G., Meng Q., Finley T., Wang T., Chen W., Ma W., Ye Q., Liu T.-Y. LightGBM: A highly efficient gradient boosting decision tree. Proceedings of the 31st International Conference on Neural Information Processing Systems.

[35] Akiba T., Sano S., Yanase T., Ohta T., Koyama M. Optuna: A Next-generation Hyperparameter Optimization Framework. Proceedings of the 25th ACM SIGKDD International Conference on Knowledge Discovery & Data Mining.

